# The Relationship of Interpersonal Conflict Handling Styles and Marital Conflicts Among Iranian Divorcing Couples

**DOI:** 10.5539/gjhs.v6n6p245

**Published:** 2014-08-15

**Authors:** Ali Navidian, Farshad Bahari, Fatihe Kermansaravi

**Affiliations:** 1 Pregnancy Health Research Center, Zahedan University of Medical Sciences, Zahedan, Iran; 2 Counseling Department of Islamic Azad University, Science and Research Arak Branch, Arak, Iran

**Keywords:** marital conflict, divorcing couples, interpersonal conflict management style

## Abstract

**Background:**

Various research studies have suggested that among other variables that couples remain married if they successfully manage their interactions (marital communication based on acceptance of individual differences, problem solving skills, forgiveness, collaborative decision making, empathy and active listening) and constructively manage conflict.

**Purpose:**

The study was aimed at examining the relation of conflict handling styles and marital conflicts among divorcing couples.

**Methods:**

As a descriptive–comparative study 60 couples out of 440 couples referred to the Crisis Intervention Center of the Isfahan Well-being Organization have selected. The tools implemented were Marital Conflicts (Barati & Sanaei, 1996) and Interpersonal Conflict Handling Styles Questionnaires (Thomas-Kilman, 1975). Their total reliabilities were, respectively, 0.74 and 0.87.

**Results:**

Findings showed that there are no significant differences among their conflict handling styles and marital conflicts. Also, there was positive correlation between avoidance and competition styles and negative one between compromise, accommodation, and cooperation styles with marital conflicts. That is, these styles reduced couples’ conflicts. Finally, wives had tendency to apply accommodation style and husbands tended to use accommodation and cooperation styles to handle their conflicts.

**Conclusions:**

It is suggested to be studied couples’ views toward their own styles to handle marital conflicts and holding training courses to orient couples with advantages and disadvantages of marital conflict handling styles.

## 1. Introduction

Conflict does happen normally in all settings such as academic, vocational, marriage, family as well in personal and collective levels (Periot & Robin, 1987; Wang, 2006). One of the most common problems among families is marital conflict. Marriage begins with the dream that only the death can separate us from each other. No couples marry with the intention of separation, but the life changes propel couples to disputing (affairs such as power, money, expectations, needs, sexual relationship, children, relatives), discord, affective separation and divorce (Navidian & Bahari, 2013). Montgomery (1989) believed that marital conflict is an interactive process in which one or both spouses feel unhappy due to some aspects of their relationships and try to resolve it any way (Hamamci, 2005). Divorce is the most common facet of severe conflict and over more than half of couples who seek to counsel, finally get divorced (Worthington, 2005).

Comstock and Sterzizweick (1990) believed that it is not absence or presence of conflict which determines the “*marriage quality*” but it is how successful to handle conflicts that determine marital relationship quality. So, the core skill in long-term commitment relationships is conflict management (Guttmann, 1994; Wilmot & Hocker, 2000). Individuals’ experiences, knowledge, beliefs, and their values offer them a variety of procedures to resolve conflicts. These procedures are named “*conflict* *solution styles*”. Conflict solution styles are, indeed, the patterned responses or sets of behaviors which people utilize them while confronting conflicts (Wilmot & Hocker, 2000).

As Grohan (1992) and Newman and Newman (1987) suggested a prerequisite of maintaining marriage is to be able to use conflict innovately. If conflict is handled constructively, development and richness of it is guaranteed and if it is handled destructively, spouses can’t stand to be merely dissatisfied with their relationships.

Miller and colleagues (1991), believe that when a discrepancy occurs between couples, they respond through one of the ways such as avoidance, surrender, uncertainly, reconciliation and participation resolution before reaching any result (reach an impasse or conflict). Kilmann and Thomas (1975) described a five styled approaches as *cooperation, accommodation, compromise, competition, and* *avoidance* based on two dimensions: self- interesting and interesting to others. Kai and Leong (2000) in their cross-cultural comparative research on conflict resolution styles showed that although a global generalization is that collectivistic societies are likely more than individualistic societies have non-confrontational approach, but they showed that individualistic societies do avoid conflicts more than collectivistic societies.

It has been appeared in most of studies that men and women have different conflict resolution styles (Kurdeck, Barril, & Watson, 1993; Mac Dual, 1990; Thomas & Kilmann, 1978). Pop and Natalya (1989) indicated that gender differences play a role in rising conflict. Men often display dominating and competitive behaviors and women present avoidant and compromising behaviors (Wang, 2006). Tannon (1994) concluded, too, in his studies that women are more likely to avoid conflict, men are more than women likely to control conversation in their favorite orientations. Women often tend to maintain their roles as “listeners” rather than “speakers”. so, they have to be settled in a weaken status to be heard (Wang, 2006).

Although, conflict handling styles are linked to gender, they may be different from one culture to other ones. Kertch, Meyer and Cohen (1992) believe that conflict handling styles are affected by cultural context. Also, Obuchi and takahashi (1994) claimed that a conflict resolution style that may be acceptable within a culture may not be acceptable in another culture. Di Cook (1995), Ferray (1993), Hope (1987) and Kagan, Night and Martishes-Romiro (1982) in their studies found that cultures have significant differences in how to approach a conflict. The Styles of conflict resolution affected by cultural context (Bartos & Wehr, 2002) and a desirable conflict resolution style in one culture may unpleasant in another (Obuchi & Takahashi, 1994). Cultures are different in their preferred forms of handling conflict (Cai & Fink, 2002). Also studies have linked culture to conflict style preference. For example, Ting-Toomey and her colleagues (1991) found respondents from China and Taiwan to be more avoiding than those from Japan, Korea, and the United States. Shaap et al. (1988) found that all conflict styles, but problem solving (cooperation) that sounds to be special styles for satisfactory marriages, have negative correlations with marital satisfaction.

Finally, Greeff and Bruyne (2000) in a study on conflict handling style and marital satisfaction using interpersonal conflict management styles (ICMS) concluded that the most common style used by males is avoidance and the least style (rather than mixed styles) was cooperation. Females report that the most common style was accommodation and the used least style was competition. The overall conclusion was that cooperation style is accompanied with the most marital satisfaction both for wives and husbands.

In the past, according to the common myth and social traditions in Iranian culture such as “in the sexual life, the women must tolerate” they had to live with their spouse lifelong. They sacrificed their marriage satisfaction for maintaining the social dignity and the satisfaction of their family-of-origin. This action causes that the divorce occurs with the sever conflicts and the interfering the law and courts as well as the continuity of life with low quality. But, now the risk of divorce has been increased because of promotion of the belief about gender equality among Iranian couples especially women and absence of essential skills for respect for freedom and disability to resolving sexual conflicts (Islami, 2009; Ahmadi, 2008).

Regarding with these findings, this research is tend to study the role of interpersonal conflict management styles (ICMS) in reducing divorcing couples’ marital conflicts who referred to Divorce Crisis Intervention Center (DCIC) by Family Court.

## 2. Methodology

Statistical population was 440 couples who due to severe marital conflict referred to Divorce Prevention Center (DPC) in Isfahan city by family court. Based on court of justice policy in Iran, all couples filing for divorce have to get advice and Consultation from Disagreement Solving Councils (DSC) including Crisis Intervention Center (CIC) of Welfare Organizations before making final decision for divorce. This is a law in Iran. We recruited these couples for our study. The sampling method was simple random. 60 couples (120 people) completed marital conflict questionnaire (MCQ) and interpersonal conflict management styles (ICMS). Marital Conflict Questionnaire (MCQ) composed of 42 items which estimate severity of marital conflicts in seven areas as follow: cooperation reduction, reduced sexual relationship, increased emotional reactions, increased personal bonding with her/his friends and family of origin, increased relationship with spouse’s friends and original family, and separating economic issues (Barati & Sanaei, 1996). This Questionnaire classifies couples’ conflict at four levels: non-conflict (ranged from 42 to 74), normal conflict (ranged from 75 to 114), and conflict higher than normal (ranged from 115 to134) and escalated conflict (ranged from135 to 210). Its reliability in this study was estimated, using Cronbach’s alpha, about 0.74. Also, Interpersonal conflict management styles (ICMS) contained 25 items which assesses five styles of interpersonal conflict handling as follow: avoidance, competition, compromise, accommodation, and cooperation. High score in each style indicates the most common used style and lowest score displays the least utilized style in resolving marital conflicts. Its reliability after deleting five items was calculated approximately 0.79.

## 3. Ethical Consideration

The participants were informed both verbally and by a written consent form attached to the questionnaire. They were assured that their participation was voluntary and anonymous. All data was handled with confidentiality. The ethical application has been approved by the ethics committee of the University of Isfahan, Iran.

## 4. Results

In this research 60 couples were studied. Significance of Levin’s test for variances homogeneity was 0.136 (df1 = 1, df2 = 58) and Levin’s statistic was 2.272 which is higher than p = 0.005. So, there are pre-requisites to use parametric tests to analyze data. Findings are presented in Tables 1-4.

**Table 1:** Levin’s test for homogeneity of variances of groups (n = 60 couples)

Variable	Levin’s statistic	Df1	Df2	Sig(2-tailed)
0.136	58	1	2.272	Marital Conflicts

**Table 2:** Results of independent t-test for checking significant difference of scores’ [Means of divorcing couples’ marital conflicts and its dimensions (n = 120)]

Reliability Statistics	Husbands		Wives		Couples		t-test	Sig (2tailed)

	Mean	SD.	Mean	SD.	Mean	SD.		
**Decreased Cooperation**	8.85	2.51	7.60	2.09	8.23	2.38	2.424	0.02
**Decreased sexual relationship**	12.85	3.52	12.10	3.04	12.48	3.29	1.019	0.31
**Increased emotional reactions**	20.68	4.79	20.65	4.61	20.66	4.67	0.024	0.98
**Increased acquirement of Child’s support**	9.93	3.09	10.45	3.49	10.19	3.28	-0.713	0.49
**Increased relatedness with one’s own relatives**	11.65	3.25	13.08	3.12	12.36	3.25	-1.998	0.49
**Decreased relatedness with spouse’s own relatives**	13.78	4.209	13.45	3.13	13.61	3.69	0.392	0.70
**Separating financial Issues**	16.93	3.48	14.98	3.35	15.95	3.536	2.551	0.01

**Total Marital Conflicts**	94.65	11.70	92.30	15.17	95.07	13.88	0.776	0.44

**Table 3:** Results of independent t-test for checking significant difference of score [Means of divorcing couples’ interpersonal conflict handling styles (n = 60)]

Gender Statistics	Husbands		Wives		couples		t-test	Sig (tailed)

	Mean	S.D	Mean	S.D	Mean	S.D		
**Avoidance**	13.32	2.69	13.08	2.61	13.48	3.10	1.597	0.11
**Competition**	6.00	1.51	5.45	1.47	5.78	1.58	-1/014	0.31
**Compromise**	12.95	3.24	12.78	2.56	13.68	2.81	0.493	0.62
**Accommodation**	17.85	3.11	18.43	2.99	18.22	3.44	-.140	0.89
**Cooperation**	17.22	3.84	16.62	3.09	17.18	3.57	1.427	**0.16**
**Total Styles**	67.33	10.94	66.37	9.02	68.35	10.88	0.854	**0.40**

**Table 4:** Correlation coefficient between Couples’ marital [Conflicts and interpersonal conflict handling styles (n = 60)]

Conflict Handle Styles	Avoid-ance	Competition	Compromise	Accommodation	Cooperation
**Decreased Cooperation**	0.43^**^	-0.72	10.271*	-0.295*	-0.395**
	0.01	0.59	0.04	0.02	0.002

**Decreased sexual relationship**	-0.58	-0.138	0.10	-0.006	-0.160
	0.66	0.30	0.94	0.96	0.22

**Increased emotional reactions**	-0.169	-0.099	0.012	-0.060	-0.293*
	0.20	0.45	0.93	0.65	0.02

**Increased Pulling in Child’s support**	0.201	-0.293*	0.144	0.296*	0.142
	0.12	0.02	0.29	0.02	0.28

**Increased relationship with one’s own relatives**	0.090	0.096	0.038	-0.031	-0.192
	0.49	0.47	0.77	0.81	0.14

**Decreased relationship with spouse’s own relatives**	0.057	0.046	0.226	0.129	0.139
	0.67	0.73	0.08	0.33	0.29

**Separating financial Issues**	-0.040	0.005	0.309*	-0.058	0.034
	0.76	0.97	0.02	0.66	0.80

Total Marital Conflicts	-0.053	-0.25	-0.147	-0.268*	-0.309*
	0.69	0.85	0.26	0.04	0.02

** Significance at 0.01-level;

* Significance at 0.05-level.

In order to determine differences between couples, independent student-t test has been used, and Pearson’s correlation statistic has been used to study the relationship between interpersonal conflict handling styles. Tables 2, 3, 4 show means and standard deviations of couples’ scores in marital conflict and marital solution styles and correlation between these two variables as well.

As it has been showed in Table 2. There are differences between couples in marital arenas of decreased cooperation, increased relatedness with one’s own relatives, and separating financial issues, respectively, in significant levels of 0.018, 0.049, and 0.013, therefore, regarding to couples’ scores’ mean in decreased cooperation dimension (8.85 for men and 8.23 for women). Increased relatedness with one’s own relatives dimension (11.65 for men and 13.08 for women) and Separating financial Issues dimension (16.93 for men and 14.98 for women) it may be said that husbands report decreased cooperation and separating financial issues more than their wives, but wives report that they had more relationship with their own friends and relatives than their husbands Although, as showed in Table 3, divorcing men reported to use accommodation-cooperation (mixed) styles, respectively with means = 17.85 and 17.22, as common conflict handling styles while confronting with interpersonal conflicts and their wives’ common style was accommodation (with mean = 18.43), but independent t-test had not shown any significant differences between couples. The least utilized style by them was competition style.

Results of Table 4 show that there are meaningful correlations between some styles of conflict handling and some areas of marital conflicts, e.g. there are correlations between avoidance style (r = -0.403, p = 0.01) and compromise style (r = -0.271, p = 0.04) with Decreased Cooperation, that is, couples who utilized these two styles to reduce their marital conflicts, reported decreased cooperativeness more. But there are relationships between styles of accommodation (r = 0.259, p = 0.02) and cooperation (r = -0.395, p = 0.02) with Decreased Cooperativeness area of marital conflicts. Regarding to negative coefficient of Decreased Cooperativeness area with accommodation & cooperation styles, it would be argued that couples who applied these two styles reported more cooperation to resolve their marital conflicts with each other.

Also, there are significant correlations among styles of competition (r = -0.395, p = 0.03) and accommodation (r = 0.296, p = 0.02) with dimension of getting child’s support, that is, couples who used competition style, move to get their children’s supports (triangulation) to face marital conflicts. Which is refers to the work of Murray Bowen. Bowen theorized that a two-person emotional system is unstable in that it forms itself into a three-person system or triangle under stress. Another finding is that there is meaningful correlation between couples’ compromise style and area of separating financial issues of marital conflicts, it means those spouses who applied this conflict solution style didn’t report to separate their earnings from each other. Also, existence of negative correlation between couples’ cooperation style and their Increased emotional reactions (r = -0.293, p = 0.02) indicates that those couples who used this style showed decreased emotional reactions. Eventually, it would be found significant correlations, respectively, among two conflict handling styles of accommodation (r = -0.268, p = 0.04) and cooperation (r = -0.309, p = 0.02) with total marital conflicts which it means couples who used these two styles reported that their total marital conflicts have been decreased.

## 5. Discussion

Based on Table 2 divorcing couples were experiencing normal marital conflicts (x = 95.07), also the comparison of couples’ scores mean showed no significant difference in total marital conflicts and its areas as well. But, as Guerin et al. (1987) and Young and Long (2007) have cited, the typical markers of level 4 of marital conflicts are: 1) Deciding to divorce, and 2) Engaging with an attorney. This is true for couples in this research, but the congruency between their attempts to get divorce and scores’ mean may be due to their low tolerances to face with marital problems or their weaken problem solving skills such as conflict solution styles. Since, although their conflicts are normal but they were experiencing it hard.

Table 3 indicates that divorcing couples both stated their rare styles were competition. And both reported their common styles as cooperation and accommodation. This finding confirms Gayle-Hackett’s views (1991) concerned to this fact that couples’ positive views could significantly modify the given response. While getting to conflicts, people tend to present themselves in a positive manner and pretend others in a negative one.

Also, this finding confirms the other research(e.g. Kretch-Meyer & Cohen, 1992; Obuchi & Takahashi, 1994; Di Cook, 1995; Hope Kagan & Zahen,1977; Kagan, Night, & Martishes-Romiro, 1982) which conjointly claimed that conflict management styles are affected by cultural contexts, as well it may not be accepted a culture’s conflict handling style by other cultures. Finally, this finding is confirms Kai and Leong (2000) who stated that the pluralistic societies more likely than individual-oriented societies have a non-confrontational approaches to conflict situations. Because Iranians are recommended a lot to be accommodated with others. Also, in eastern cultures, included Iranian culture, it has been recommended to consider pluralism prior than individualism. The typical marker is belongingness (Selleh-e-Rahem) which is raised from Islamic culture.

Another finding is this point that the divorcing husbands the mixed style (mixture of accommodating – cooperating styles) is concomitant with Greeff and Bruyne (2000) who showed that those who use mixed styles lead their partners to be confused, so they dissatisfy with marriages. In the accommodating–cooperating mixed style, the individual is flexible and seeking for creative solutions, “give and take”, behavior exchange (cooperating) and in some cases, they have to surrender passively either with happy or unwillingness; this occurs as compassionate and accountability toward spouse (accommodating).

The last finding suggest that cooperation and accommodation styles have generally negative correlations with marital conflicts, which is the same as Shaap et al. (1988) who concluded all conflict solution styles but cooperation have negative relations with marital conflicts. Meanwhile, although accommodation style has been shown to have negative correlation with marital satisfaction, but in this study it was effective in reducing couples’ conflicts. This finding has cultural and religious explanations, because Islamic texts always order women to be obliged to their men.

Limitations of the present study

One limitation of the present study is that the couples in our sample exhibited wide variation in age, duration of marriage, reasons for claiming divorce, education in non-academic and academic studies, intrapersonal problems and interpersonal problems. In addition, the current study was limited in terms of the urgent conditions of some of the couples requesting divorce, and the obligation of the crisis intervention center (CIC) to respond to the court’s instructions.

## 6. Conclusion

In brief, regarding to these findings we suggest couples to be oriented with their own interpersonal conflict handling styles which may be lead to marital conflicts, as well, advantages and disadvantages of each styles could be explained.
